# (*Z*)-2,3-Di­chloro-1,4-bis­(4-chloro­phen­yl)but-2-ene-1,4-dione

**DOI:** 10.1107/S1600536814015463

**Published:** 2014-07-11

**Authors:** Ram K. Tittal, Satish Kumar, R. N. Ram

**Affiliations:** a Department of Chemistry, Indian Institute of Technology Delhi, Hauzkhas, New Delhi 110 016, India; b Department of Chemistry, St. Stephen’s College, University Enclave, Delhi 110 007, India

**Keywords:** crystal structure

## Abstract

The title compound, C_16_H_8_Cl_4_O_2_, crystallizes with two independent mol­ecules in the asymmetric unit. Both mol­ecules have a *Z* conformation around the central double bond and they show significantly different C—C—C—O torsion angles between the aromatic ring and the carbonyl group [30.1 (7) and 3.9 (7)° in one molecule and 23.5 (7) and 9.3 (8)° in the other]. The crystal packing shows short halogen Cl⋯O [3.003 (5) and 3.246 (4) Å] and Cl⋯Cl [3.452 (2) Å] contacts and aromatic C—H⋯Cl and C—H⋯O inter­actions link the molecules, resulting in chains propogating along [100]. The crystal structure also features π–π stacking inter­actions between aromatic units of the two independent mol­ecules, with a centroid–centroid distance of 3.9264 (6) Å.

## Related literature   

For general background and details of the synthesis, see: Clark (2002 ▶); Martin *et al.* (1985 ▶); Matyjaszewski & Xia (2001 ▶); Ram & Charles (1999 ▶); Ram & Kumar (2008 ▶); Ram & Tittal (2014*a* ▶,*b* ▶); Ram & Manoj (2008 ▶); Ram & Meher (2003 ▶); Ram *et al.* (2007 ▶); Tomislav & Matyjaszewski (2008 ▶). For halogen-bond inter­actions, see: Agarwal *et al.* (2014 ▶); Gonnade *et al.* (2008 ▶); Pedireddi *et al.* (1992 ▶). For short aromatic inteactions, see: Warad *et al.* (2013 ▶).
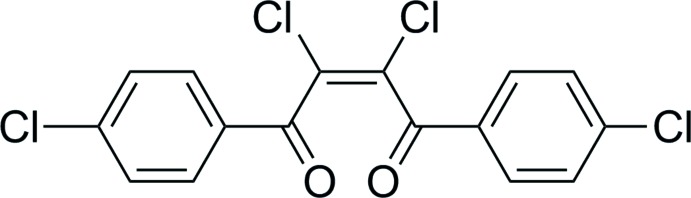


## Experimental   

### 

#### Crystal data  

C_16_H_8_Cl_4_O_2_
*M* *_r_* = 374.02
Orthorhombic, 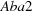

*a* = 19.065 (2) Å
*b* = 28.668 (4) Å
*c* = 11.8800 (14) Å
*V* = 6493.1 (14) Å^3^
*Z* = 16
Mo *K*α radiation
μ = 0.73 mm^−1^
*T* = 273 K
0.37 × 0.28 × 0.20 mm

#### Data collection  

Bruker SMART APEX CCD detector diffractometer
Absorption correction: multi-scan (*SADABS*; Bruker, 2000 ▶) *T*_min_ = 0.782, *T*_max_ = 0.863
31827 measured reflections
6044 independent reflections
5194 reflections with *I* > 2σ(*I*)
*R*_int_ = 0.068

#### Refinement  

*R*[*F* ^2^ > 2σ(*F* ^2^)] = 0.065
*wR*(*F* ^2^) = 0.145
*S* = 1.13
6044 reflections
397 parameters
1 restraint
H-atom parameters constrained
Δρ_max_ = 0.31 e Å^−3^
Δρ_min_ = −0.22 e Å^−3^
Absolute structure: Flack (1983 ▶), 1939 Friedel pairs
Absolute structure parameter: 0.08 (7)

### 

Data collection: *SMART* (Bruker, 2000 ▶); cell refinement: *SAINT* (Bruker, 2000 ▶); data reduction: *SAINT*; program(s) used to solve structure: *SHELXS97* (Sheldrick, 2008 ▶); program(s) used to refine structure: *SHELXL97* (Sheldrick, 2008 ▶); molecular graphics: *Mercury* (Macrae *et al.*, 2008 ▶) and *SHELXTL* (Sheldrick, 2008 ▶); software used to prepare material for publication: *publCIF* (Westrip, 2010 ▶), *PLATON* (Spek, 2009 ▶) and *SHELXTL*.

## Supplementary Material

Crystal structure: contains datablock(s) I, New_Global_Publ_Block. DOI: 10.1107/S1600536814015463/fj2678sup1.cif

Structure factors: contains datablock(s) I. DOI: 10.1107/S1600536814015463/fj2678Isup2.hkl

Supporting information file. DOI: 10.1107/S1600536814015463/fj2678Isup3.cdx

Supporting information file. DOI: 10.1107/S1600536814015463/fj2678Isup4.cml

CCDC reference: 1011687

Additional supporting information:  crystallographic information; 3D view; checkCIF report

## Figures and Tables

**Table 1:** Hydrogen-bond geometry (Å, °)

*D*—H⋯*A*	*D*—H	H⋯*A*	*D*⋯*A*	*D*—H⋯*A*
C2—H2⋯Cl7	0.93	2.74	3.160 (5)	109
C28—H28⋯Cl2	0.93	2.72	3.191 (5)	112
C3—H3⋯O1^i^	0.93	2.55	3.290 (6)	137
C5—H5⋯O3^ii^	0.93	2.75	3.418 (6)	129
C6—H6⋯O3^ii^	0.93	2.91	3.502 (6)	122
C13—H13⋯O2^iii^	0.93	2.45	3.302 (7)	152
C29—H29⋯Cl8^iv^	0.93	2.81	3.645 (5)	149

Symmetry codes: (i) 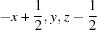; (ii) 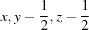; (iii) 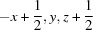; (iv) 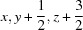.

**Fig. 1.**
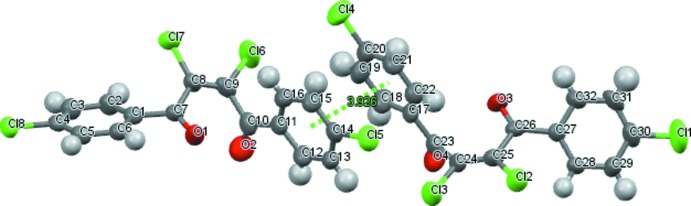
The molecular structure of the title compound, with atom labels and 50% probability displacement ellipsoids for non-H atoms showing π-π stacking interactions.

**Fig. 2.**
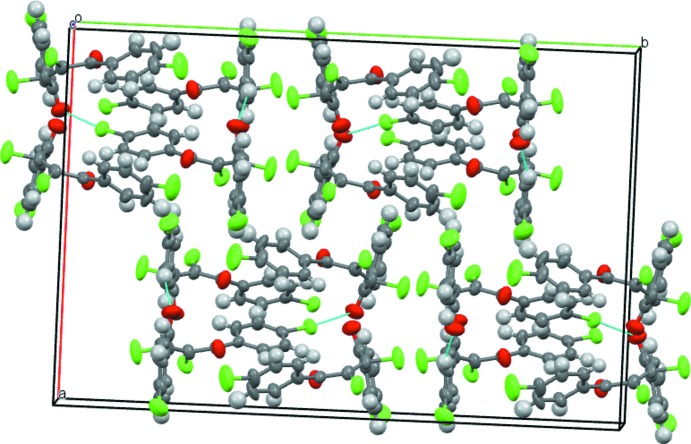
The packing diagram of the title compound showing short intermolecular halogen bond Cl···O interactions.

**Fig. 3.**
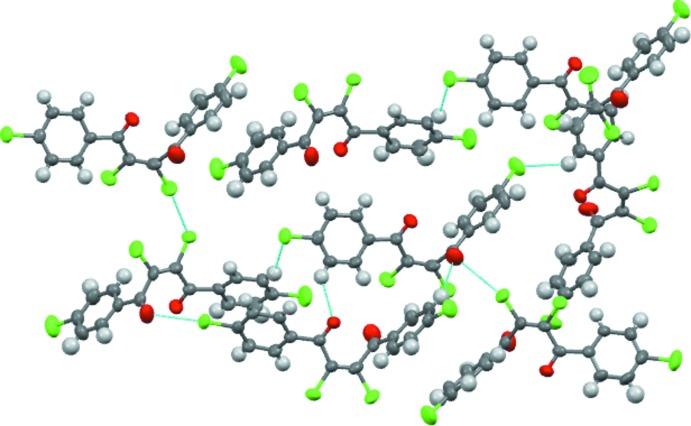
Structure of the title compound showing Cl···Cl, O···Cl, C—H···Cl and C—H···O interactions.

## supplementary crystallographic information

### S2. Structural commentary

Free radical reactions are intimately involved in the chemistry of tri­chloro­methyl compounds. Generation of free radicals from tri­chloro­methyl group by homolysis of a C—Cl bond is relatively easy. Free radicals can easily be generated by the action of UV-light, radical initiators or redox active metal salts or its complexes. Considerable amount of information is available in the literature on radical reactions involving tri­chloro­methyl group containing compounds. For example, the radical generated by reaction of a tri­chloro­methyl group substituted compound under non reducing condition with CuCl or its complexes with bpy or with other bi- or tridentate tertiary amine ligands readily undergo inter­molecular (Martin *et al.*, 1985) or intra­molecular (Clark, 2002), (Ram & Kumar, 2008) addition/cyclization on to a suitably substituted carbon-carbon double bond. The formation of mono-and/or di-reduction product are also reported under non reducing conditions along with cyclization products (Ram *et al.*, 2007). Such radicals also acts as radical initiator in atom transfer radical polymerization reactions (Tomislav & Matyjaszewski, 2008), (Matyjaszewski & Xia , 2001). However, if the carbon-carbon double bond in such radical centre is replaced by any weak or relatively better leaving group at the β-position of the radical centre, it underwent predominantly rearrangement and/or fragmentation by the inter­mediate formation of contact ion pair (Ram & Meher, 2003). It is worthwhile to mention that 2,2,2-tri­chloro­ethyl­alkyl ethers and tri­chloro­methyl carbinols having no suitably located carbon-carbon double bond or a leaving group β-position to the tri­chloro­methyl carbon underwent 1,2-H shift under similar conditions through the inter­mediacy of a copper-carbenoid species (Ram & Charles, 1999), (Ram & Manoj, 2008). In this context, we have decided to explore the behavior of the radicals derived from tri­chloro­methyl compounds which neither contains any suitably located carbon-carbon double bond nor any leaving group or any hydrogen atom at the β-position of the radical centre so as to restrict the above transformations i.e. inter­molecular or intra­molecular addition; ATRP; rearrangement and/or fragmentation or 1,2-H shift. The major product obtained under such reaction conditions is reported here. The asymmetric unit (Fig. 1) consists of the two formula units of the compound. Each formula unit adopts Z conformation about the C=C bond: C_8_=C_9_ and C_24_=C_25_. The aromatic ring of two units are nearly coplanar with a dihedral angle of 12.73° (C_12_—C_15_—C_21_—C_18_). A centroid to centroid distance of 3.9264 (6) Å between aromatic units of two independent molecules present in the asymmetric unit is observed indicating the presence of π–π stacking inter­actions (Fig. 1). The structure is stabilized by short inter­molecular C—H···Cl [3.160 (5), 3.191 (5) and 3.645 (5)Å], C—H···O [3.290 (6), 3.418 (6), 3.502 (6) and 3.302 (7)Å] inter­actions [Warad *et al.* (2013)] (Fig. 2). In addition, the crystal packing also features short Cl···O {O_2_···Cl_3_ [3.003 (5)] Å and O_4_···Cl_8_ [3.246 (4) Å]} and Cl_2_···Cl_6_ [3.452 (2)Å] halogen bond inter­actions (Fig. 3) (Gonnade *et al.*, 2008), (Pedireddi *et al.*, 1992), Agarwal *et al.* (2014).

### S5. Synthesis and crystallization

A two-neck round bottom flask fitted with a rubber septum was charged with CuCl (0.8 g, 0.008mol), 2,2'-bi­pyridine (1.25 g, 0.008 mol). Nitro­gen was introduced into the flask followed by addition of 15 mL dry DCE or benzene into the flask to ensure the formation of the brown colored CuCl-bpy complex. To the reaction flask a solution of the 2,2,2-tri­chloro-1-(4-chloro-phenyl)-ethanone(0.004 mol) in dry DCE or benzene (5 mL) was added with the with the help of a syringe and the reaction mixture was heated to reflux with stirring under a slow and continuous flow of nitro­gen. After the completion of the reaction as indicated by TLC (1-2 h), the reaction mixture was cooled and filtered through a celite pad. The filtrate was evaporated under reduced pressure on a rotary evaporator and purified by column chromatography using silica gel as the solid support. A solution of *n*-hexane and ethyl­acetate was used as the solvent for elution to get 1 in 52 or 60 % isolated yields in DCE or benzene respectively. Suitable crystals were obtained from chloro­form/henxane. Melting point 110 °C.

### S6. Refinement

Crystal data, data collection and structure refinement details are summarized in Table 1. All H atoms were placed at their ideal position with C—H = 0.93 A°.

### Crystal data

**Table:**

C_16_H_8_Cl_4_O_2_	*D*_x_ = 1.530 Mg m^−^^3^
*M**_r_* = 374.02	Melting point: 383 K
Orthorhombic, *A**b**a*2	Mo *K*α radiation, λ = 0.71073 Å
Hall symbol: A 2 -2ac	Cell parameters from 5754 reflections
*a* = 19.065 (2) Å	θ = 3.2–26.1°
*b* = 28.668 (4) Å	µ = 0.73 mm^−^^1^
*c* = 11.8800 (14) Å	*T* = 273 K
*V* = 6493.1 (14) Å^3^	Block, colourless
*Z* = 16	0.37 × 0.28 × 0.20 mm
*F*(000) = 3008	

### Data collection

**Table:**

Bruker SMART APEX CCD detector diffractometer	6044 independent reflections
Radiation source: fine-focus sealed tube	5194 reflections with *I* > 2σ(*I*)
Graphite monochromator	*R*_int_ = 0.068
φ and ω scans	θ_max_ = 25.5°, θ_min_ = 1.4°
Absorption correction: multi-scan (*SADABS*; Bruker, 2000)	*h* = −23→23
*T*_min_ = 0.782, *T*_max_ = 0.863	*k* = −34→34
31827 measured reflections	*l* = −14→14

### Refinement

**Table:**

Refinement on *F*^2^	Secondary atom site location: difference Fourier map
Least-squares matrix: full	Hydrogen site location: inferred from neighbouring sites
*R*[*F*^2^ > 2σ(*F*^2^)] = 0.065	H-atom parameters constrained
*wR*(*F*^2^) = 0.145	*w* = 1/[σ^2^(*F*_o_^2^) + (0.0741*P*)^2^] where *P* = (*F*_o_^2^ + 2*F*_c_^2^)/3
*S* = 1.13	(Δ/σ)_max_ < 0.001
6044 reflections	Δρ_max_ = 0.31 e Å^−^^3^
397 parameters	Δρ_min_ = −0.22 e Å^−^^3^
1 restraint	Absolute structure: Flack (1983), 1939 Friedel pairs
Primary atom site location: structure-invariant direct methods	Absolute structure parameter: 0.08 (7)

### Special details

**Table:**

Geometry. All e.s.d.'s (except the e.s.d. in the dihedral angle between two l.s. planes) are estimated using the full covariance matrix. The cell e.s.d.'s are taken into account individually in the estimation of e.s.d.'s in distances, angles and torsion angles; correlations between e.s.d.'s in cell parameters are only used when they are defined by crystal symmetry. An approximate (isotropic) treatment of cell e.s.d.'s is used for estimating e.s.d.'s involving l.s. planes.
Refinement. Refinement of *F*^2^ against ALL reflections. The weighted *R*-factor *wR* and goodness of fit *S* are based on *F*^2^, conventional *R*-factors *R* are based on *F*, with *F* set to zero for negative *F*^2^. The threshold expression of *F*^2^ > σ(*F*^2^) is used only for calculating *R*-factors(gt) *etc*. and is not relevant to the choice of reflections for refinement. *R*-factors based on *F*^2^ are statistically about twice as large as those based on *F*, and *R*- factors based on ALL data will be even larger.

### Fractional atomic coordinates and isotropic or equivalent isotropic displacement parameters (Å^2^)

**Table:**

	*x*	*y*	*z*	*U*_iso_*/*U*_eq_	
C1	0.1802 (2)	0.18216 (14)	0.0202 (4)	0.0441 (10)	
C2	0.2138 (3)	0.18813 (16)	−0.0820 (4)	0.0560 (12)	
H2	0.2257	0.2180	−0.1056	0.067*	
C3	0.2296 (2)	0.15075 (16)	−0.1490 (4)	0.0553 (12)	
H3	0.2540	0.1549	−0.2160	0.066*	
C4	0.2091 (2)	0.10726 (15)	−0.1158 (4)	0.0479 (11)	
C5	0.1760 (2)	0.09998 (15)	−0.0130 (4)	0.0491 (11)	
H5	0.1631	0.0701	0.0094	0.059*	
C6	0.1627 (2)	0.13761 (17)	0.0547 (4)	0.0493 (11)	
H6	0.1418	0.1332	0.1246	0.059*	
C7	0.1635 (2)	0.22072 (15)	0.0995 (4)	0.0458 (10)	
C8	0.1486 (3)	0.26865 (17)	0.0565 (4)	0.0520 (12)	
C9	0.1600 (3)	0.30479 (17)	0.1200 (5)	0.0584 (13)	
C10	0.1935 (3)	0.30333 (16)	0.2379 (5)	0.0543 (12)	
C11	0.1455 (2)	0.30832 (14)	0.3351 (4)	0.0471 (11)	
C12	0.1746 (3)	0.31256 (18)	0.4422 (5)	0.0612 (14)	
H12	0.2231	0.3141	0.4499	0.073*	
C13	0.1333 (3)	0.3145 (2)	0.5364 (5)	0.0653 (15)	
H13	0.1531	0.3172	0.6077	0.078*	
C14	0.0618 (3)	0.31252 (17)	0.5223 (4)	0.0595 (14)	
C15	0.0316 (3)	0.30947 (19)	0.4183 (5)	0.0645 (14)	
H15	−0.0169	0.3092	0.4113	0.077*	
C16	0.0730 (3)	0.30681 (17)	0.3243 (5)	0.0586 (13)	
H16	0.0526	0.3040	0.2535	0.070*	
C17	0.1304 (3)	0.45602 (16)	0.5455 (4)	0.0494 (11)	
C18	0.1596 (3)	0.4566 (2)	0.4390 (5)	0.0661 (15)	
H18	0.2072	0.4631	0.4313	0.079*	
C19	0.1208 (3)	0.4480 (2)	0.3454 (5)	0.0743 (16)	
H19	0.1416	0.4484	0.2746	0.089*	
C20	0.0506 (3)	0.43870 (19)	0.3565 (5)	0.0653 (14)	
C21	0.0195 (3)	0.43733 (19)	0.4611 (5)	0.0681 (15)	
H21	−0.0280	0.4306	0.4682	0.082*	
C22	0.0595 (3)	0.44599 (18)	0.5550 (5)	0.0604 (13)	
H22	0.0388	0.4451	0.6259	0.072*	
C23	0.1738 (3)	0.46744 (16)	0.6438 (4)	0.0543 (12)	
C24	0.1466 (3)	0.45532 (16)	0.7593 (4)	0.0552 (12)	
C25	0.1170 (2)	0.48387 (16)	0.8325 (4)	0.0514 (12)	
C26	0.1019 (2)	0.53398 (15)	0.8002 (4)	0.0462 (10)	
C27	0.0991 (2)	0.57237 (16)	0.8868 (4)	0.0459 (11)	
C28	0.1346 (2)	0.57170 (15)	0.9873 (4)	0.0466 (11)	
H28	0.1594	0.5451	1.0085	0.056*	
C29	0.1339 (3)	0.60992 (17)	1.0567 (4)	0.0584 (13)	
H29	0.1595	0.6097	1.1234	0.070*	
C30	0.0961 (3)	0.64784 (18)	1.0281 (5)	0.0671 (15)	
C31	0.0593 (3)	0.64972 (18)	0.9285 (6)	0.0768 (17)	
H31	0.0335	0.6761	0.9097	0.092*	
C32	0.0614 (3)	0.61211 (17)	0.8578 (4)	0.0597 (13)	
H32	0.0373	0.6131	0.7897	0.072*	
Cl1	0.09338 (14)	0.69524 (6)	1.11846 (19)	0.1247 (8)	
Cl2	0.08763 (8)	0.46534 (4)	0.96328 (11)	0.0696 (4)	
Cl3	0.16115 (10)	0.39746 (4)	0.79049 (14)	0.0897 (5)	
Cl4	−0.00090 (11)	0.42888 (7)	0.23804 (16)	0.1048 (6)	
Cl5	0.00905 (9)	0.31287 (6)	0.64225 (14)	0.0877 (5)	
Cl6	0.14301 (12)	0.36143 (5)	0.08012 (14)	0.0981 (6)	
Cl7	0.11250 (8)	0.27538 (5)	−0.07659 (12)	0.0712 (4)	
Cl8	0.22478 (7)	0.05907 (4)	−0.20286 (12)	0.0635 (3)	
O1	0.1630 (2)	0.21511 (12)	0.1997 (3)	0.0657 (10)	
O2	0.2555 (2)	0.30041 (15)	0.2467 (4)	0.0835 (12)	
O3	0.0931 (2)	0.54208 (12)	0.7004 (3)	0.0653 (9)	
O4	0.2329 (2)	0.48314 (14)	0.6368 (4)	0.0807 (12)	

### Atomic displacement parameters (Å^2^)

**Table:**

	*U*^11^	*U*^22^	*U*^33^	*U*^12^	*U*^13^	*U*^23^
C1	0.039 (2)	0.040 (2)	0.053 (3)	−0.0054 (18)	−0.001 (2)	0.005 (2)
C2	0.072 (3)	0.038 (2)	0.059 (3)	−0.005 (2)	0.006 (3)	0.009 (2)
C3	0.063 (3)	0.051 (3)	0.052 (3)	−0.005 (2)	0.010 (2)	0.005 (2)
C4	0.039 (2)	0.049 (3)	0.056 (3)	0.007 (2)	−0.009 (2)	−0.004 (2)
C5	0.048 (3)	0.039 (2)	0.061 (3)	−0.0011 (19)	0.002 (2)	0.007 (2)
C6	0.048 (3)	0.054 (3)	0.045 (3)	0.004 (2)	0.000 (2)	0.003 (2)
C7	0.050 (3)	0.043 (2)	0.044 (3)	0.003 (2)	−0.003 (2)	0.005 (2)
C8	0.065 (3)	0.044 (3)	0.047 (3)	0.011 (2)	0.003 (2)	0.004 (2)
C9	0.066 (3)	0.044 (3)	0.065 (3)	0.014 (2)	0.016 (3)	0.014 (2)
C10	0.062 (3)	0.037 (2)	0.063 (3)	−0.009 (2)	0.012 (3)	−0.004 (2)
C11	0.050 (3)	0.037 (2)	0.055 (3)	−0.0023 (19)	0.000 (2)	−0.006 (2)
C12	0.047 (3)	0.068 (3)	0.069 (4)	0.000 (2)	−0.008 (3)	−0.016 (3)
C13	0.066 (4)	0.075 (4)	0.054 (3)	0.025 (3)	−0.012 (3)	−0.020 (3)
C14	0.070 (3)	0.052 (3)	0.056 (3)	0.018 (2)	0.005 (3)	−0.009 (2)
C15	0.051 (3)	0.082 (4)	0.061 (3)	0.002 (3)	0.004 (3)	−0.011 (3)
C16	0.056 (3)	0.067 (3)	0.053 (3)	0.004 (2)	−0.008 (2)	−0.008 (2)
C17	0.054 (3)	0.041 (3)	0.052 (3)	−0.002 (2)	−0.003 (2)	−0.012 (2)
C18	0.056 (3)	0.075 (4)	0.067 (4)	−0.015 (3)	0.005 (3)	−0.012 (3)
C19	0.087 (4)	0.085 (4)	0.051 (3)	−0.006 (3)	0.005 (3)	−0.010 (3)
C20	0.076 (4)	0.062 (3)	0.058 (3)	0.001 (3)	−0.008 (3)	−0.011 (3)
C21	0.053 (3)	0.069 (3)	0.082 (4)	−0.012 (3)	−0.001 (3)	−0.010 (3)
C22	0.065 (3)	0.064 (3)	0.053 (3)	−0.009 (3)	0.006 (3)	0.004 (3)
C23	0.063 (3)	0.043 (3)	0.058 (3)	0.004 (2)	−0.001 (3)	−0.008 (2)
C24	0.071 (3)	0.039 (3)	0.055 (3)	0.004 (2)	−0.012 (2)	−0.009 (2)
C25	0.060 (3)	0.048 (3)	0.047 (3)	−0.004 (2)	−0.007 (2)	0.012 (2)
C26	0.043 (2)	0.048 (3)	0.047 (3)	−0.0041 (19)	−0.009 (2)	0.009 (2)
C27	0.051 (3)	0.049 (3)	0.038 (2)	0.006 (2)	0.009 (2)	0.005 (2)
C28	0.053 (3)	0.045 (2)	0.041 (2)	0.009 (2)	0.001 (2)	0.006 (2)
C29	0.079 (3)	0.053 (3)	0.043 (3)	0.003 (3)	−0.003 (3)	−0.002 (2)
C30	0.099 (4)	0.046 (3)	0.057 (3)	0.014 (3)	0.024 (3)	0.001 (2)
C31	0.096 (4)	0.048 (3)	0.086 (4)	0.036 (3)	0.004 (3)	0.006 (3)
C32	0.064 (3)	0.051 (3)	0.064 (3)	0.016 (2)	−0.008 (2)	0.004 (3)
Cl1	0.204 (2)	0.0658 (10)	0.1046 (15)	0.0227 (12)	0.0211 (15)	−0.0310 (10)
Cl2	0.1076 (11)	0.0476 (6)	0.0537 (8)	−0.0028 (7)	0.0051 (7)	0.0125 (6)
Cl3	0.1506 (14)	0.0439 (7)	0.0746 (10)	0.0210 (8)	−0.0108 (11)	−0.0020 (7)
Cl4	0.1133 (14)	0.1214 (15)	0.0795 (11)	−0.0006 (11)	−0.0345 (10)	−0.0287 (10)
Cl5	0.0900 (10)	0.1093 (13)	0.0638 (9)	0.0236 (9)	0.0217 (8)	−0.0129 (9)
Cl6	0.1784 (18)	0.0426 (7)	0.0734 (10)	0.0264 (9)	0.0176 (11)	0.0112 (7)
Cl7	0.0909 (10)	0.0691 (8)	0.0534 (7)	0.0193 (7)	−0.0084 (7)	0.0110 (6)
Cl8	0.0727 (8)	0.0537 (7)	0.0641 (8)	0.0115 (6)	−0.0026 (7)	−0.0138 (6)
O1	0.101 (3)	0.052 (2)	0.044 (2)	0.0158 (18)	0.0010 (19)	0.0062 (16)
O2	0.056 (2)	0.114 (3)	0.080 (3)	−0.010 (2)	0.011 (2)	−0.006 (2)
O3	0.095 (3)	0.055 (2)	0.046 (2)	0.0059 (18)	−0.0172 (19)	0.0064 (16)
O4	0.063 (2)	0.097 (3)	0.082 (3)	−0.016 (2)	−0.002 (2)	−0.028 (2)

### Geometric parameters (Å, º)

**Table:**

C1—C6	1.382 (6)	C17—C18	1.382 (7)
C1—C2	1.383 (7)	C17—C22	1.386 (7)
C1—C7	1.487 (6)	C17—C23	1.468 (7)
C2—C3	1.368 (7)	C18—C19	1.359 (8)
C2—H2	0.9300	C18—H18	0.9300
C3—C4	1.365 (6)	C19—C20	1.372 (8)
C3—H3	0.9300	C19—H19	0.9300
C4—C5	1.390 (7)	C20—C21	1.377 (8)
C4—Cl8	1.751 (5)	C20—Cl4	1.738 (6)
C5—C6	1.369 (7)	C21—C22	1.374 (8)
C5—H5	0.9300	C21—H21	0.9300
C6—H6	0.9300	C22—H22	0.9300
C7—O1	1.201 (5)	C23—O4	1.216 (6)
C7—C8	1.493 (6)	C23—C24	1.508 (7)
C8—C9	1.299 (7)	C24—C25	1.322 (7)
C8—Cl7	1.736 (5)	C24—Cl3	1.722 (5)
C9—C10	1.541 (8)	C25—C26	1.514 (6)
C9—Cl6	1.722 (5)	C25—Cl2	1.735 (5)
C10—O2	1.190 (6)	C26—O3	1.220 (6)
C10—C11	1.479 (7)	C26—C27	1.507 (7)
C11—C16	1.389 (7)	C27—C28	1.373 (7)
C11—C12	1.393 (7)	C27—C32	1.390 (6)
C12—C13	1.369 (8)	C28—C29	1.371 (7)
C12—H12	0.9300	C28—H28	0.9300
C13—C14	1.375 (7)	C29—C30	1.348 (7)
C13—H13	0.9300	C29—H29	0.9300
C14—C15	1.366 (8)	C30—C31	1.377 (9)
C14—Cl5	1.744 (6)	C30—Cl1	1.733 (6)
C15—C16	1.369 (8)	C31—C32	1.367 (8)
C15—H15	0.9300	C31—H31	0.9300
C16—H16	0.9300	C32—H32	0.9300
			
C6—C1—C2	119.1 (4)	C18—C17—C22	118.0 (5)
C6—C1—C7	116.6 (4)	C18—C17—C23	119.9 (5)
C2—C1—C7	124.3 (4)	C22—C17—C23	122.1 (5)
C3—C2—C1	121.0 (4)	C19—C18—C17	121.9 (5)
C3—C2—H2	119.5	C19—C18—H18	119.1
C1—C2—H2	119.5	C17—C18—H18	119.1
C4—C3—C2	119.0 (5)	C18—C19—C20	119.2 (6)
C4—C3—H3	120.5	C18—C19—H19	120.4
C2—C3—H3	120.5	C20—C19—H19	120.4
C3—C4—C5	121.4 (4)	C19—C20—C21	120.8 (5)
C3—C4—Cl8	120.1 (4)	C19—C20—Cl4	120.3 (5)
C5—C4—Cl8	118.5 (4)	C21—C20—Cl4	118.9 (5)
C6—C5—C4	118.8 (4)	C22—C21—C20	119.3 (5)
C6—C5—H5	120.6	C22—C21—H21	120.4
C4—C5—H5	120.6	C20—C21—H21	120.4
C5—C6—C1	120.6 (4)	C21—C22—C17	120.8 (5)
C5—C6—H6	119.7	C21—C22—H22	119.6
C1—C6—H6	119.7	C17—C22—H22	119.6
O1—C7—C1	122.0 (4)	O4—C23—C17	123.4 (5)
O1—C7—C8	117.4 (4)	O4—C23—C24	117.8 (5)
C1—C7—C8	120.6 (4)	C17—C23—C24	118.6 (4)
C9—C8—C7	120.3 (4)	C25—C24—C23	127.1 (4)
C9—C8—Cl7	120.4 (4)	C25—C24—Cl3	121.6 (4)
C7—C8—Cl7	119.3 (4)	C23—C24—Cl3	111.3 (3)
C8—C9—C10	125.1 (4)	C24—C25—C26	120.1 (4)
C8—C9—Cl6	124.1 (4)	C24—C25—Cl2	122.5 (4)
C10—C9—Cl6	110.7 (4)	C26—C25—Cl2	117.1 (4)
O2—C10—C11	123.6 (5)	O3—C26—C27	121.3 (4)
O2—C10—C9	119.5 (5)	O3—C26—C25	116.9 (4)
C11—C10—C9	116.8 (4)	C27—C26—C25	121.8 (4)
C16—C11—C12	118.9 (5)	C28—C27—C32	118.8 (5)
C16—C11—C10	122.7 (5)	C28—C27—C26	124.4 (4)
C12—C11—C10	118.4 (4)	C32—C27—C26	116.6 (4)
C13—C12—C11	121.4 (5)	C29—C28—C27	120.4 (4)
C13—C12—H12	119.3	C29—C28—H28	119.8
C11—C12—H12	119.3	C27—C28—H28	119.8
C12—C13—C14	118.0 (5)	C30—C29—C28	119.9 (5)
C12—C13—H13	121.0	C30—C29—H29	120.1
C14—C13—H13	121.0	C28—C29—H29	120.1
C15—C14—C13	122.0 (5)	C29—C30—C31	121.4 (5)
C15—C14—Cl5	119.8 (4)	C29—C30—Cl1	119.5 (5)
C13—C14—Cl5	118.2 (4)	C31—C30—Cl1	119.1 (4)
C14—C15—C16	119.9 (5)	C32—C31—C30	118.8 (5)
C14—C15—H15	120.0	C32—C31—H31	120.6
C16—C15—H15	120.0	C30—C31—H31	120.6
C15—C16—C11	119.8 (5)	C31—C32—C27	120.6 (5)
C15—C16—H16	120.1	C31—C32—H32	119.7
C11—C16—H16	120.1	C27—C32—H32	119.7
			
C6—C1—C2—C3	0.1 (7)	C22—C17—C18—C19	0.4 (9)
C7—C1—C2—C3	177.8 (4)	C23—C17—C18—C19	−177.5 (5)
C1—C2—C3—C4	2.8 (8)	C17—C18—C19—C20	0.4 (9)
C2—C3—C4—C5	−3.6 (7)	C18—C19—C20—C21	−1.0 (9)
C2—C3—C4—Cl8	176.9 (4)	C18—C19—C20—Cl4	178.5 (5)
C3—C4—C5—C6	1.3 (7)	C19—C20—C21—C22	0.8 (9)
Cl8—C4—C5—C6	−179.2 (3)	Cl4—C20—C21—C22	−178.7 (4)
C4—C5—C6—C1	1.7 (7)	C20—C21—C22—C17	0.0 (8)
C2—C1—C6—C5	−2.4 (7)	C18—C17—C22—C21	−0.6 (8)
C7—C1—C6—C5	179.8 (4)	C23—C17—C22—C21	177.3 (5)
C6—C1—C7—O1	30.1 (7)	C18—C17—C23—O4	9.3 (8)
C2—C1—C7—O1	−147.6 (5)	C22—C17—C23—O4	−168.5 (5)
C6—C1—C7—C8	−151.7 (4)	C18—C17—C23—C24	−165.2 (5)
C2—C1—C7—C8	30.5 (7)	C22—C17—C23—C24	17.0 (7)
O1—C7—C8—C9	24.3 (7)	O4—C23—C24—C25	84.3 (7)
C1—C7—C8—C9	−153.9 (5)	C17—C23—C24—C25	−100.9 (6)
O1—C7—C8—Cl7	−153.0 (4)	O4—C23—C24—Cl3	−93.7 (5)
C1—C7—C8—Cl7	28.8 (6)	C17—C23—C24—Cl3	81.1 (5)
C7—C8—C9—C10	4.4 (8)	C23—C24—C25—C26	5.1 (8)
Cl7—C8—C9—C10	−178.3 (4)	Cl3—C24—C25—C26	−177.1 (3)
C7—C8—C9—Cl6	−178.6 (4)	C23—C24—C25—Cl2	179.9 (4)
Cl7—C8—C9—Cl6	−1.3 (7)	Cl3—C24—C25—Cl2	−2.3 (6)
C8—C9—C10—O2	80.1 (7)	C24—C25—C26—O3	26.5 (7)
Cl6—C9—C10—O2	−97.3 (5)	Cl2—C25—C26—O3	−148.6 (4)
C8—C9—C10—C11	−103.2 (6)	C24—C25—C26—C27	−152.6 (5)
Cl6—C9—C10—C11	79.5 (5)	Cl2—C25—C26—C27	32.3 (5)
O2—C10—C11—C16	−173.1 (5)	O3—C26—C27—C28	−152.0 (5)
C9—C10—C11—C16	10.3 (6)	C25—C26—C27—C28	27.0 (7)
O2—C10—C11—C12	3.9 (7)	O3—C26—C27—C32	23.5 (7)
C9—C10—C11—C12	−172.7 (4)	C25—C26—C27—C32	−157.5 (4)
C16—C11—C12—C13	1.0 (7)	C32—C27—C28—C29	−1.2 (7)
C10—C11—C12—C13	−176.1 (5)	C26—C27—C28—C29	174.2 (4)
C11—C12—C13—C14	−0.4 (8)	C27—C28—C29—C30	2.3 (8)
C12—C13—C14—C15	−1.2 (8)	C28—C29—C30—C31	−1.7 (9)
C12—C13—C14—Cl5	177.7 (4)	C28—C29—C30—Cl1	178.0 (4)
C13—C14—C15—C16	2.2 (9)	C29—C30—C31—C32	0.1 (9)
Cl5—C14—C15—C16	−176.7 (4)	Cl1—C30—C31—C32	−179.6 (5)
C14—C15—C16—C11	−1.5 (8)	C30—C31—C32—C27	1.0 (9)
C12—C11—C16—C15	0.0 (7)	C28—C27—C32—C31	−0.5 (8)
C10—C11—C16—C15	177.0 (5)	C26—C27—C32—C31	−176.3 (5)

### Hydrogen-bond geometry (Å, º)

**Table:**

*D*—H···*A*	*D*—H	H···*A*	*D*···*A*	*D*—H···*A*
C2—H2···Cl7	0.93	2.74	3.160 (5)	109
C28—H28···Cl2	0.93	2.72	3.191 (5)	112
C3—H3···O1^i^	0.93	2.55	3.290 (6)	137
C5—H5···O3^ii^	0.93	2.75	3.418 (6)	129
C6—H6···O3^ii^	0.93	2.91	3.502 (6)	122
C13—H13···O2^iii^	0.93	2.45	3.302 (7)	152
C29—H29···Cl8^iv^	0.93	2.81	3.645 (5)	149

Symmetry codes: (i) −*x*+1/2, *y*, *z*−1/2; (ii) *x*, *y*−1/2, *z*−1/2; (iii) −*x*+1/2, *y*, *z*+1/2; (iv) *x*, *y*+1/2, *z*+3/2.
